# A Pilot Comparative Study between Creatinine- and Cystatin-C-Based Equations to Estimate GFR and Kidney Ultrasound Percentiles in Children with Congenital Anomalies of the Kidney and Urinary Tract

**DOI:** 10.3390/diagnostics14100994

**Published:** 2024-05-10

**Authors:** Ruxandra Maria Steflea, Ramona Stroescu, Mihai Gafencu, Emil Robert Stoicescu, Raluca Isac, Ioana-Cristina Olariu, Andrada Mara Micsescu-Olah, Septimiu Radu Susa, Mircea Murariu, Gabriela Doros

**Affiliations:** 1 Department of Pediatrics, “Victor Babes” University of Medicine and Pharmacy, Eftimie Murgu Square 2, 300041 Timisoara, Romania; steflea.ruxandra@umft.ro (R.M.S.); stroescu.ramona@umft.ro (R.S.); isac.raluca@umft.ro (R.I.); olariu.cristina@umft.ro (I.-C.O.); andrada.micsescu-olah@umft.ro (A.M.M.-O.); doros.gabriela@umft.ro (G.D.); 2 “Louis Turcanu” Emergency Hospital for Children, Iosif Nemoianu Street 2, 300011 Timisoara, Romania; mircea.murariu@umft.ro; 3 Department of Radiology and Medical Imaging, “Victor Babes” University of Medicine and Pharmacy, Eftimie Murgu Square No. 2, 300041 Timisoara, Romania; stoicescu.emil@umft.ro; 4 Research Center for Pharmaco-Toxicological Evaluations, “Victor Babes” University of Medicine and Pharmacy, Eftimie Murgu Square No. 2, 300041 Timisoara, Romania; 5 Field of Applied Engineering Sciences, Specialization Statistical Methods and Techniques in Health and Clinical Research, Faculty of Mechanics, “Politehnica” University, Mihai Viteazu Boulevard No. 1, 300222 Timisoara, Romania; 6 Doctoral School, “Victor Babes” University of Medicine and Pharmacy, Eftimie Murgu Square 2, 300041 Timisoara, Romania; septimiu.susa@umft.ro

**Keywords:** pediatric nephrology, congenital anomalies, glomerular filtration rate (eGFR), bedside Schwartz equation, cystatin C based full age spectrum formula, CKid U25, kidney ultrasound percentiles, urinary tract abnormalities, kidney imaging, kidney function assessment

## Abstract

Congenital anomalies affecting the kidneys present significant challenges in pediatric nephrology, needing precise methods for assessing renal function and guiding therapeutic intervention. Bedside Schwartz formula with the cystatin-C-based Full Age Spectrum formula and Chronic Kidney Disease in Children (CKiD) U 25 formula used in estimating glomerular filtration rate (eGFR) and also to assess if the eGFR in association with kidney length percentiles can be a monitoring parameter for the progression of chronic kidney disease in children with congenital anomalies of the kidney and urinary tract (CAKUT). A total of 64 pediatric patients (median age at diagnostic was 12 months with an interquartile range of 2 to 60) were diagnosed with congenital anomalies in the kidney and urinary tract between June 2018 and May 2023 at “Louis Turcanu” Emergency Hospital for Children in Timisoara, Romania. Baseline characteristics, CAKUT types, associated pathologies, CKD staging, and eGFR using creatinine and cystatin C were analyzed. The mean age at the moment of examination was 116.50 months; (65, 180). Chronic kidney disease staging revealed a predominance of patients in CKD stages G1 and A1. Analysis of eGFR methods revealed a small mean difference between eGFR estimated by creatinine and cystatin C, with a moderate-strong positive correlation observed between the eGFR and ultrasound parameters. Using cystatin-C-based formulas for eGFR, in conjunction with ultrasound measurements, may offer reliable insights into renal function in pediatric patients with congenital anomalies affecting the kidney and urinary tract. However, the economic aspect must be taken into consideration because cystatin C determination is approximately eight times more expensive than that of creatinine. An interdisciplinary approach is crucial for managing patients with CAKUT.

## 1. Introduction

Congenital anomalies affecting the kidneys and urinary tract (CAKUT) represent a significant challenge in pediatric nephrology, demanding thorough and precise methods for assessing renal function and guiding therapeutic interventions [1,2]. Among the arsenal of diagnostic tools available, the bedside Schwartz equation, cystatin C clearance, and kidney ultrasound percentiles have emerged as prominent contenders, each offering distinct advantages and considerations in the evaluation of renal health in children with congenital anomalies [3,4,5].

The original Schwartz equations, initially developed in 1976 and subsequently refined, have long been a cornerstone in estimating glomerular filtration rate (eGFR) in pediatric populations. In 2009, Schwartz et al. developed a revised estimated GFR formula using data from a cohort of children in the Chronic Kidney Disease in Children (CKiD) study [5,6,7].

Based on serum creatinine (S_Cr_) levels and patient demographics, these equations provide a practical means of assessing renal function, particularly in clinical settings where direct measurement of GFR is challenging. However, their reliance on creatinine levels, which can be influenced by factors such as muscle mass and diet, has prompted scrutiny regarding their accuracy, especially in certain patient populations [6,8].

In contrast, cystatin C, a low molecular weight protein produced at a constant rate by nucleated cells, has garnered attention as a potentially superior marker of renal function [9,10]. Unlike creatinine, cystatin C is less influenced by extrarenal factors, offering a more reliable eGFR, particularly in pediatric patients with altered muscle mass or diet restrictions [6,10,11]. Consequently, cystatin C clearance has emerged as a promising alternative or adjunct to traditional creatinine-based equations, especially in populations where accuracy is paramount, such as children with congenital anomalies affecting renal function [9,10].

Using data from the CKiD study a complementary estimating equation based on cysC was developed: the “CKiD under 25 (U25)” eGFR equations (where 25 refers to age in years) [12].

Moreover, advancements in imaging technology have facilitated the use of kidney ultrasound percentiles as a complementary tool in the assessment of renal morphology and function [13,14]. Ultrasounds offer the benefit of diagnosing and monitoring medical conditions in children without the use of radiation. This is particularly useful when assessing soft tissues such as the kidney or changes in the bone. In the advanced stages of CKD, there is a strong association with mineral-bone disease and a high risk of fractures [15,16,17]. By comparing kidney size and echogenicity to normative data, clinicians can identify structural abnormalities and monitor disease progression in pediatric patients with congenital anomalies [18]. However, the interpretation of ultrasound findings requires meticulous attention to patient age, body size, and developmental stage, highlighting the need for standardized protocols and reference values in pediatric nephrology practice [13,19].

In this comparative analysis, we aim to elucidate the strengths, limitations, and practical implications of using eGFR calculated using either bedside Schwartz formula, cystatin-C-based Full Age Spectrum formula, or CkiDU25 formula in association with kidney ultrasound percentiles in the evaluation of renal health in children with congenital anomalies. Our future goal is to help clinicians and researchers make informed decisions about renal function assessment and treatment for vulnerable populations based on the existing literature and clinical insights.

## 2. Methodology

### 2.1. Study Design

This study employed a retrospective observational design, using data collected from medical records of patients with congenital anomalies in the kidney and urinary tract (CAKUT).

Ethical approval was obtained from the institutional review board of the “Louis Turcanu” Emergency Hospital for Children Timisoara (no. 118/25 November 2023) and the “Victor Babes” University of Medicine and Pharmacy Timisoara (no. 68/3 October 2022).

Patient confidentiality and privacy were maintained throughout the study, with all data de-identified prior to analysis.

Informed consent was obtained in all cases throughout the admission chart.

### 2.2. Patient Population

The study included pediatric patients diagnosed with CAKUT.

CAKUT was defined as a group of developmental disorders that affect the kidneys (including renal agenesis, size, position, and morphology) and outflow tracts, such as the ureters, bladder, and urethra. Ureteropelvic junction obstruction, vesicoureteral reflux, duplex collecting system, megaureter, and posterior urethral valves represented the outflow abnormalities [1,20].

Chronic kidney disease (CKD) is defined as the presence of abnormalities in kidney structure or function for at least three months, which can have implications for overall health. Structural abnormalities detected by imaging included our patients in the CKD category in correlation with their eGFR [21].

CKD staging was as per Kidney Disease: Improving Global Outcomes (KDIGO) guidelines, as follows:

○ GFR Category: G1- ≥ 90 mL/min/1.73 m^2^, G2- 60–89 mL/min/1.73 m^2^, G3a- 45–59 mL/min/1.73 m^2^, G3b- 30–44 mL/min/1.73 m^2^, G4- 15–29 mL/min/1.73 m^2^, G5 <15 mL/min/1.73 m^2^;
○ Albuminuria using albumin to creatinine ratio (ACR- approximate equivalent) category: A1- < 3 mg/mmol or <30 mg/g, A2- 3–30 mg/mmol or 30–300 mg/g, A3 >30 mg/mmol or >300 mg/g [22].

Patients were identified through electronic medical records from the “Louis Turcanu” Emergency Hospital for Children Timisoara between June 2018 and May 2023.

The inclusion criteria consisted of children between the ages of 0 and 18 with CAKUT and CKD who were admitted to the hospital.

The exclusion criteria included:

- Children with urinary tract infections during the evaluation;
- Children in whom serum cystatin C was not obtained at the same time as the Scr.

**Figure 1:**
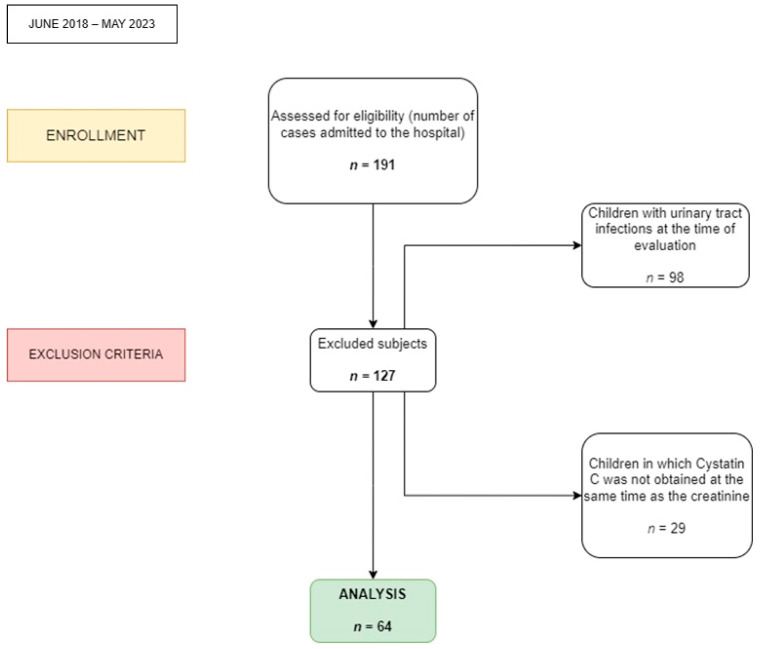
Flow diagram of the progress through the stages (enrollment, exclusion criteria, and analysis of the children).

Figure 1 provides a comprehensive overview of the procedures involved in the recruitment, assignment, and evaluation of patients.

### 2.3. Data Collection

Demographic data, including age, sex, environment of origin, weight, and height were extracted from patient records. Clinical data such as diagnosis, comorbidities, and medication history were recorded.

### 2.4. Laboratory Assessment

Laboratory measurements included serum creatinine and serum cystatin C. Serum samples were analyzed using a Cobas Integra^®^ 400 Plus analyzer from Roche Diagnostics (CH-6343 Rotkreuz, Basel, Switzerland).

Serum samples were obtained by centrifugation at 4000 rotations/min for 8 min. Afterward, the serum was processed as per laboratory protocol.

Urine samples were also collected from each patient and screened for proteinuria.

Abdominal ultrasounds were performed using an Esaote MyLab™ Seven unit (ESAOTE S.p.A., Genova, Italy). The MyLab™ Seven, manufactured by Esaote, follows the Medical Device Directive (MDD) 93\42\EEC. In accordance with this directive, Esaote has classified these units as Class IIa devices. The studies were conducted by trained pediatric radiologists or a qualified pediatric nephrologist with more than five years experience. The patients were in a supine position. After kidney identification, it was visualized along its longitudinal axis crossing through the kidney hilum. The measured values of kidney length in millimeters were recorded for each patient.

After obtaining the kidney length, the kidney length percentiles and z scores were established in correlation with the patient’s age at the time of the measurement [14]. The z-score is a statistical measurement that tells how far a data point is from the mean of a group of values, in terms of standard deviations. A z-score can be positive or negative, depending on whether the data point is above or below the mean. A z-score of 0 means that the data point is equal to the mean. A z-score can be used to place a data point on a normal distribution curve [23].

### 2.5. Estimation of Glomerular Filtration Rate (eGFR)

eGFR was provided using the following Schwartz formula based on serum creatinine levels and patient height: 0.413 × height/S_Cr_, where height is measured in centimeters and S_Cr_ in mg/dl [5].

Cystatin-C-based GFR estimation was performed using the Full Age Spectrum (FAS) (Cystatin C Equation 2017) [24,25].

CKid U25 equation is KCysC/SerumCystatinC, where Age-based variables are assigned values by this ordered logic:

- If age is <12 years and sex is female, then K_Cr_ = 36.1 × 1.008 (age—12) and K_CysC_ = 79.9 × 1.004 (age—12);
- Else if age is <12 years and sex is male, then K_Cr_ = 39 × 1.008 (age—12) and K_CysC_ = 87.2 × 1.011 (age—15);
- Else if age is <15 years and sex is female, then K_Cr_ = 36.1 × 1.023 (age—12) and K_CysC_ = 79.9 × 0.974 (age—12);
- Else if age is <15 years and sex is male, then K_Cr_ = 39 × 1.045 (age—12) and K_CysC_ = 87.2 × 1.011 (age—15);
- Else if age is <18 years and sex is female, then K_Cr_ = 36.1 × 1.023 (age—12) and K_CysC_ = 79.9 × 0.974 (age—12);
- Else if age is <18 years and sex is male, hen K_Cr_ = 39 × 1.045 (Age—12) and K_CysC_ = 87.2 × 0.960 (Age—15);
- Else if age is ≤25 years and sex is female, then K_Cr_ = 41.4 and K_CysC_ = 68.3;
- Else if age is ≤25 years and sex is male, then K_Cr_ = 50.8 and K_CysC_ = 77.1 [12,26].

Comparative analyses were conducted to assess the concordance and discordance between the eGFR obtained from the methods described above.

### 2.6. Statistical Analysis

Statistical analyses were performed using MedCalc^®^ Statistical Software version 22.017 (MedCalc Software Ltd., Ostend, Belgium; https://www.medcalc.org; accessed on 15 January 2024).

The identification data, along with clinical and paraclinical parameters, were meticulously documented within a secure computerized database using Microsoft Excel version 2312 (Build 17126.20132), released on 9 January 2024.

Descriptive statistics were used to summarize patient demographics, clinical characteristics, and laboratory findings.

The agreement between the eGFR obtained from the revised bedside Schwartz formula and the FAS Cystatin C equation was assessed using correlation coefficients and Bland–Altman analysis.

The Shapiro–Wilk test was used to assess the distribution of the plotted data, and non-parametric statistical methods were employed because of a significant deviation from a normal distribution.

Measures of central tendency were calculated utilizing medians and the interquartile range [IQR] for variables that did not meet parametric assumptions.

The research utilized Pearson correlation coefficients (r) and linear regression to assess the strength and direction of the associations between ultrasound parameters and eGFR. The Pearson correlation coefficient, often denoted by r, is a key statistical measure used to assess the strength and direction of the linear relationship between two variables.

In order to incorporate demographic variables into the analysis of correlations, we chose to divide the males based on an age threshold of 144 months and the females based on an age threshold of 120 months. The selection of these values was based on their growth and pubertal development [27].

## 3. Results

### 3.1. Baseline Characteristics

Of the 64 children, 38 were male, which accounts for 59.38%. A total of 35 individuals originated from metropolitan regions, accounting for 54.69%. The median age at diagnosis was 12 months, with an interquartile range of 2 to 60. Anomalies were detected at birth or during prenatal care, as the minimum age for diagnosis was 0. The highest age at diagnosis was 205 months.

Table 1 displays the baseline characteristics of the population under study.

Negative z scores indicate that weight and height were below the mean of a distribution. In other words, it suggested that the value was lower than the average or typical value in the dataset. Negative z scores were indicative of observations that were on the lower end of the distribution, representing values that fall farther from the mean in the negative direction on the standard normal distribution curve.

### 3.2. Types of CAKUT

The types of CAKUT identified in our cohort are presented in Table 2. The anomaly with the highest occurrence was renal pelvis and ureter malformations at 59.38%. This could imply that this anomaly is more common in the population studied and may require more attention in terms of research and treatment strategies. It is also important to consider that the percentages for right, left, and bilateral vesicoureteral reflux are expressed from the patients with vesicoureteral reflux, not the total sample. Similarly, compensatory hypertrophy and reflux nephropathy are secondary to other conditions. These factors should be taken into account when interpreting the data.

### 3.3. Chronic Kidney Disease Staging

The majority of our patients were included in the mild to moderate stages of CKD (84.38%), and only 10 patients have severe CKD needing more complex treatment, including continuous renal replacement therapy.

Table 3 displays the incidence of chronic kidney stage among the children under analysis.

### 3.4. eGFR Using Serum Creatinine and Cystatin C

**Figure 2:**
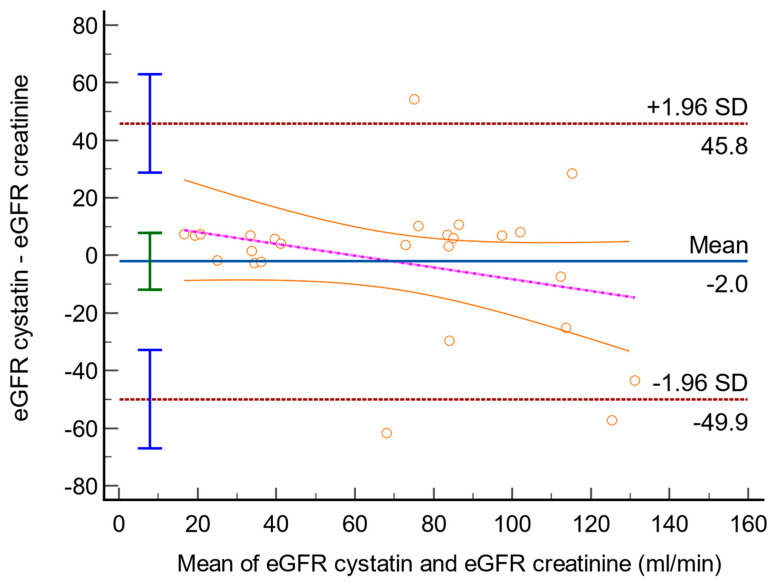
Bland–Altman plot with the following parameters: line of equality (red dotted line), 95% CI of mean difference (green interval), 95% CI of limits of the upper and lower limit of agreement (blue intervals), and regression line of differences (purple dotted line) with 95% CI (orange lines). The orange circles represent plot data from the mean eGFR cystatin and eGFR creatinine (*X*-axis) and the difference between them (*Y*-axis) from the same patient. GFR_Cystatin C = eGFR determined using FAS Cystatin C Equation. GFR_ creatinine = eGFR determined using bedside Schwartz formula.

The arithmetic mean difference between the two methods was −2.03, with a 95% confidence interval ranging from −11.90 to 7.83. The *p*-value for the null hypothesis was 0.67, suggesting no significant difference between the two methods. The lower and upper limits of the differences were −49.91 and 45.84, respectively, with their 95% confidence intervals ranging from −67.00 to −32.83 and 28.76 to 62.93, respectively. The regression equation derived from the data was y = 12.12 − 0.20x. The β_0_ and β_1_ of the regression equation were 12.12 and −0.20, respectively, with *p*-values of 0.25 and 0.13, showing that neither β_0_ nor β_1_ are significantly different from zero at the 5% level. The 95% confidence intervals for β_0_ and β_1_ were −9.27 to 33.52 and −0.47 to 0.07, respectively. These findings suggest that there is no significant difference between the two methods based on the sample data. The Bland–Altman plot is presented in Figure 2.

**Figure 3:**
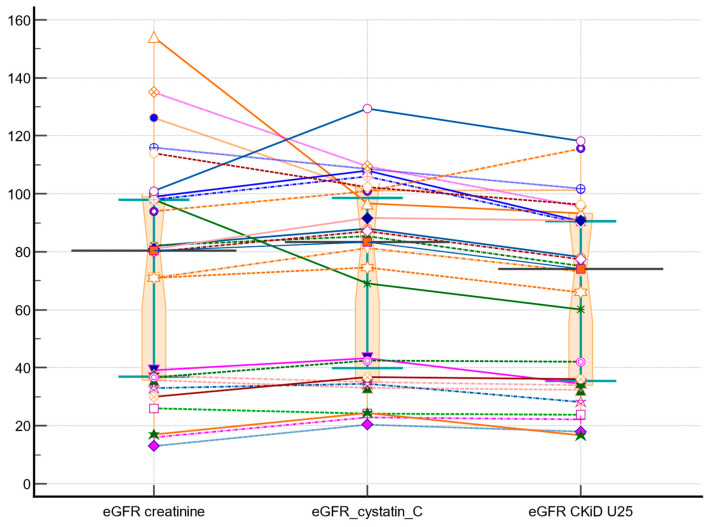
Dot-and-line diagram illustrating the relationship between variables (eGFR creatinine, eGFR cystatin C, eGFR CKID U25): box-and-whisker plot with notches for pairwise comparison, scatter plot showing all data points with a connecting line between them. Each patient is represented by distinct symbols and colors that indicate the estimated values for GFR using various approaches. The connecting lines between them serve to distinguish the variations in the methods employed.

A dot-and-line diagram illustrating the relationship between variables (eGFR creatinine, eGFR cystatin C, and eGFR CKiD U25) is shown in Figure 3. The test resulted in a Friedman statistic (F) of 13.85 with degrees of freedom 1 (DF1) of 2 and degrees of freedom 2 (DF2) of 50. The *p*-value was less than 0.001, indicating a statistically significant difference among the three methods. Multiple comparisons revealed that each method was significantly different from the other two, as indicated by their mean ranks and the minimum required difference of mean rank (0.45). Specifically, eGFR creatinine had a mean rank of 2.03, eGFR Cystatin C had a mean rank of 2.57, and eGFR CKiD U25 had a mean rank of 1.38. This suggests that the three methods provide different estimates of eGFR, and the choice of method could significantly impact the results.

**Figure 4:**
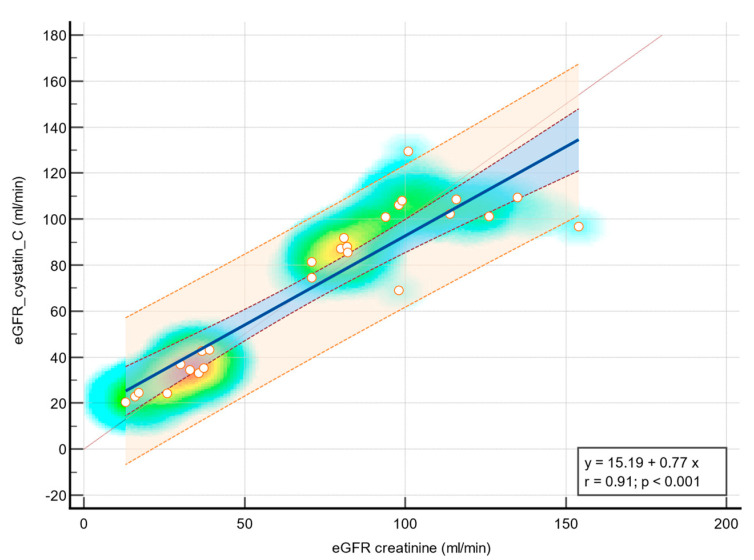
Very strong positive linear association between eGFR creatinine and eGFR cystatin C—scatter diagram and regression line with heat map, 95% confidence interval curve (blue area), 95% prediction interval curve (orange area). The background color of the heat map indicates density of points, suggesting clusters of observations. The red color indicates a high concentration of points, yellow shows a moderate concentration of points, and blue indicates a low concentration of points. The orange circles represent the correspondence between plot data from the eGFR creatinine (*X*-axis) and eGFR cystatin (*Y*-axis) from the same patient.

The coefficient of determination (R^2^) is 0.82, indicating that approximately 82.88% of the variation in eGFR cystatin C can be explained by eGFR creatinine. The residual standard deviation is 14.55. The regression equation is given by y = 15.19 + 0.77x, where the β_0_ is 15.19 (*p* = 0.01) and the β_1_ is 0.77 (*p* < 0.001). The analysis of variance shows a significant F-ratio of 116.16 (*p* < 0.001), indicating a significant relationship between the variables. Figure 4 presents the very strong correlation between eGFR creatinine and eGFR cystatin C as a scatter diagram with a heat map and line of equality.

### 3.5. Correlation between eGFR and Ultrasound Finding

The median dimension of the right kidney was 8.15 cm with an interquartile range between 6.50 cm and 9.95 cm. The median dimension of the left kidney was 9 cm with an IQR between 7.18 cm and 11.00 cm.

The median percentile of the right kidney was 29 with IQR between 1 and 88.50. The median percentile of the left kidney was 52 with IQR between 0 and 97. The median z score for the right kidney was −0.32; (−1.78, 1.36), as the z score for the left kidney was 0.07; (−2.40, 1.86).

The median z score for the right kidney being −0.32 indicates that, on average, the measurements for the right kidney are slightly below the mean of the distribution. This suggests that the values for the right kidney tend to be lower than the average measurements across the dataset.

**Figure 5:**
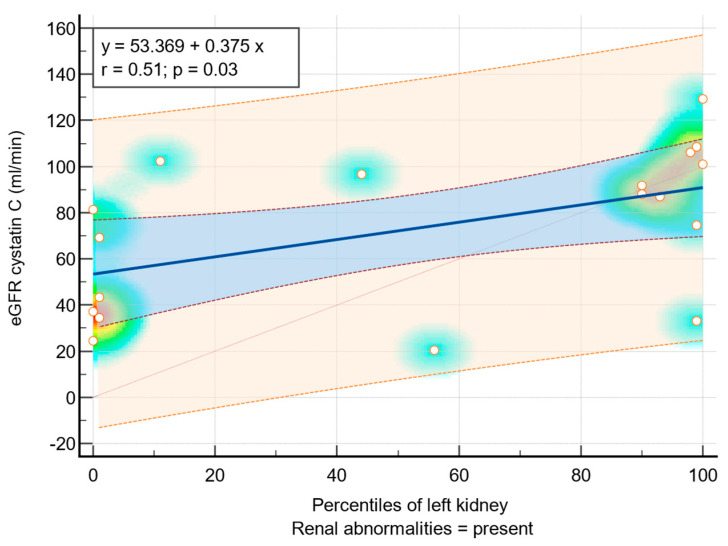
Moderate positive linear association between eGFR cystatin C and percentiles of the left kidney of patients with renal abnormalities—scatter diagram and regression line with heat map, 95% confidence interval curve (blue area), 95% prediction interval curve (orange area). The background color of the heat map shows density of points, suggesting clusters of observations. The red color shows a high concentration of points, yellow indicates a moderate concentration of points, and blue indicates a low concentration of points. The orange circles represent the correspondence between plot data from the eGFR creatinine (*X*-axis) and percentiles of the left kidney of patients with renal abnormalities (*Y*-axis) from the same patient.

According to Pearson correlation (r), the following correlations were reported between eGFR and ultrasound findings:

- r = 0.51, *p* = 0.03 between eGFR cystatin C and percentiles of the left kidney based on the kidney dimension of individuals with renal abnormalities—Figure 5;
- r = 0.48, *p* = 0.04 between eGFR cystatin C and z score of the left kidney based on the kidney dimension of individuals with renal abnormalities;
- r = 0.41, *p* = 0.08 between eGFR CKiD U25 and percentiles of the left kidney based on the kidney dimension of individuals with renal abnormalities;
- r = 0.48, *p* = 0.004 between eGFR creatinine and z score of the right kidney based on the kidney dimension of individuals with renal abnormalities.

According to the demographic factors (gender and age), the following correlations were found between eGFR and ultrasound parameters:

- r = −0.56, *p* = 0.07 between eGFR cystatin C and z score of the right kidney based on the kidney dimension of boys with age < 144 months;
- r = −0.54, *p* = 0.06 between eGFR creatinine and z score of the left kidney based on the kidney dimension of girls with age < 120 months;
- r = 0.39, *p* = 0.23 between eGFR cystatin C and percentiles of the left kidney based on the kidney dimension of boys with age < 144 months.

## 4. Discussion

The presented results provide an insight into the baseline characteristics of a pediatric population affected by CAKUT who were admitted to the “Louis Turcanu” Emergency Hospital for Children Timisoara. The male population represented 59.38%, and 56.7% of the patients were from urban areas. In comparison with other studies, the gender distribution and regional origin within this cohort appear consistent with general demographic trends [1,3,28,29,30]. However, the median age at diagnosis and examination, along with the wide interquartile range, suggest variability in the timing of anomaly detection and clinical assessment, which may differ from findings in other populations; each country has different health care systems, the number of antenatal scans as per protocol varies and the addressability of an ultrasound depends on the socio-economic status of the country [1,28]. Furthermore, the reported weight and height metrics offer insight into the physical characteristics of the study population, which may vary across different cohorts based on factors such as geographic location, ethnicity, and socio-economic status [31,32,33].

It is important to compare the different types of CAKUT and the diseases they cause with those from other studies in order to understand how common these conditions are and how important they are in clinical practice. In the same way, the high rates of renal malformations (51.56%) and renal pelvis and ureter malformations (59.38%) are in line with previous research that shows these problems are common in people born with kidney problems. However, the prevalence of vesicoureteral reflux at 37.5% and its subtypes (right, left, and bilateral reflux) may vary compared to other studies due to differences in patient demographics and study methodologies [34]. Furthermore, the prevalence of other pathologies such as renal hypoplasia, compensatory renal hypertrophy, and ureteropelvic junction stenosis falls within the expected range based on the existing literature [34].

The study conducted by Pottel et al. found that the Full Age Spectrum equations, specifically FAScysC and FAScombi, showed superior performance in estimating GFR across all age groups. In children and adolescents, FAScysC and FAScombi outperformed the Caucasian Asian Paediatric Adult Cohort equation and the ScysC-based Schwartz equation. In adults, FAScysC performed equally well as the Chronic Kidney Disease Epidemiology Collaboration equation (CKD-EPIcysC), and FAScombi was on par with the combined CKD-EPI equation. In older adults, FAScysC was superior to CKD-EPIcysC, and FAScombi outperformed the combined CKD-EPI equation. The FAS equations were found to be applicable to all ages and for all recommended renal biomarkers and their combinations [25].

The Bland–Altman plot (Figure 2) shows that there is no significant difference between the Cystatin C and Creatinine methods, as the 95% confidence interval for the mean difference lies within the range of −11.90 to 7.83, and the *p*-value for the null hypothesis is 0.67. This suggests that the two methods are comparable in estimating GFR. In contrast, the Friedman test (Figure 3) reveals a statistically significant difference among the three methods, indicating that each method provides a distinct estimate of GFR. The coefficient of determination (R^2^) of 0.82 suggests that approximately 82.88% of the variation in eGFR cystatin C can be explained by eGFR creatinine, indicating a strong positive correlation between the two methods. Overall, the results suggest that while Cystatin C and Creatinine methods are comparable in estimating GFR, they differ significantly from the CKiD U25 method, and the choice of method could significantly impact the results. These findings contribute to the understanding of eGFR methods and their applicability in pediatric patients with congenital renal anomalies [6,13,18,35].

Most patients (62.5%) fall into CKD stage G1, indicating mild impairment, while fewer individuals are classified into more advanced stages, such as CKD G2-G5. Additionally, the majority of patients (92.18%) are categorized into albuminuria stages A1, reflecting normal to mildly increased levels of urinary albumin excretion. These findings are similar to others and underscore the importance of early detection and intervention to prevent the progression of CKD in pediatric patients with CAKUT, highlighting the need for tailored management strategies to optimize outcomes and mitigate long-term complications [36,37,38].

The study reveals distinct correlations between various parameters, such as eGFR cystatin C and ultrasound findings, including percentiles and z scores of kidney dimensions. Moderate positive correlations between eGFR measured by cystatin C and ultrasound parameters (percentiles of the left kidney, z score of the left kidney) highlight the potential utility of cystatin C clearance as a reliable indicator of renal function in this context [6,37]. Furthermore, the observed correlations between eGFR measured by cystatin C and both percentiles and z scores of kidney dimensions underscore the interplay between renal function and structural characteristics, emphasizing the importance of comprehensive evaluation approaches [39]. 

Based on demographic factors, crrelations between eGFR and kidney dimensions were examined by age and gender. In boys < 144 months old, eGFR (cystatin C) correlated negatively with the right kidney’s dimension z score, and in girls < 120 months old, eGFR (creatinine) correlated negatively with the left kidney’s dimension z score. Boys < 144 months old showed a weak positive correlation between eGFR (cystatin C) and the left kidney’s dimension percentiles. These results highlight age and gender considerations in assessing kidney health.

The financial aspect necessitates consideration because the cystatin C serum determination technique is approximately eight times more costly than that of creatinine. Our research indicates that eGFR, in conjunction with renal ultrasonography, continues to be a valuable and cost-effective approach for monitoring patients with CAKUT.

Several limitations should be acknowledged in this study. Firstly, its retrospective observational design inherently introduces potential biases and limitations due to reliance on historical data. Additionally, the relatively small sample size of the study population may restrict the generalizability of the findings to a broader pediatric cohort with congenital renal anomalies. Moreover, the study’s reliance on data from a single institution may limit the diversity of the patient population, potentially overlooking variations in demographics and clinical characteristics present in different healthcare settings. Additionally, the study did not investigate the potential impact of confounding variables, such as comorbidities or medication history, on the correlations observed between eGFR and ultrasound findings.

Contemporary literature reviews suggested that improving patient-specific risk stratification in CKD can be achieved by focusing on markers reflecting the mechanism of injury [40,41]. Neutrophil-gelatinase-associated lipocalin (NGAL), interleukin 18 (IL-18), and liver-type fatty-acid-binding protein (L-FABP) are identified as potential biomarkers of tubular injury, with NGAL particularly promising due to its established diagnostic and prognostic roles in obstructive hydronephrosis and other CAKUT [33,35,36]. NGAL, a protein produced by activated neutrophils, exhibits complex activities beyond its antibacterial function and is implicated in the early detection of acute kidney injury [42,43,44]. Beta trace protein (BTP), β2-microglobulin (B2M), and Klotho are also explored as biomarkers for assessing renal function, with BTP and B2M showing correlations with traditional markers of CKD and Klotho exhibiting inconsistent results [41,45,46]. Considering the multifactorial nature of CKD progression, a panel of biomarkers may offer a more comprehensive approach for predicting development and evaluating outcomes of the disease [41]. Our future research will mostly focus on NGAL, as it has already been established in CAKUT.

In terms of future directions, prospective studies with larger sample sizes and longer follow-up periods are called for to overcome the limitations associated with retrospective designs and small sample sizes. Additionally, comparative effectiveness research could shed light on the relative advantages and disadvantages of various GFR estimation techniques, imaging modalities, and therapeutic interventions. There is also a need for research focused on finding novel biomarkers of renal function and disease progression to enhance diagnostic accuracy and prognostic capabilities in pediatric patients with congenital renal anomalies. Furthermore, interventional trials evaluating the efficacy of medical, surgical, and lifestyle interventions in managing renal anomalies would provide valuable insights into optimal treatment approaches. Lastly, health services research investigating healthcare delivery models, access to care, and disparities in outcomes among pediatric patients with congenital renal anomalies could inform efforts to optimize healthcare delivery and improve equity in access to care for this vulnerable population.

**Figure 6:**
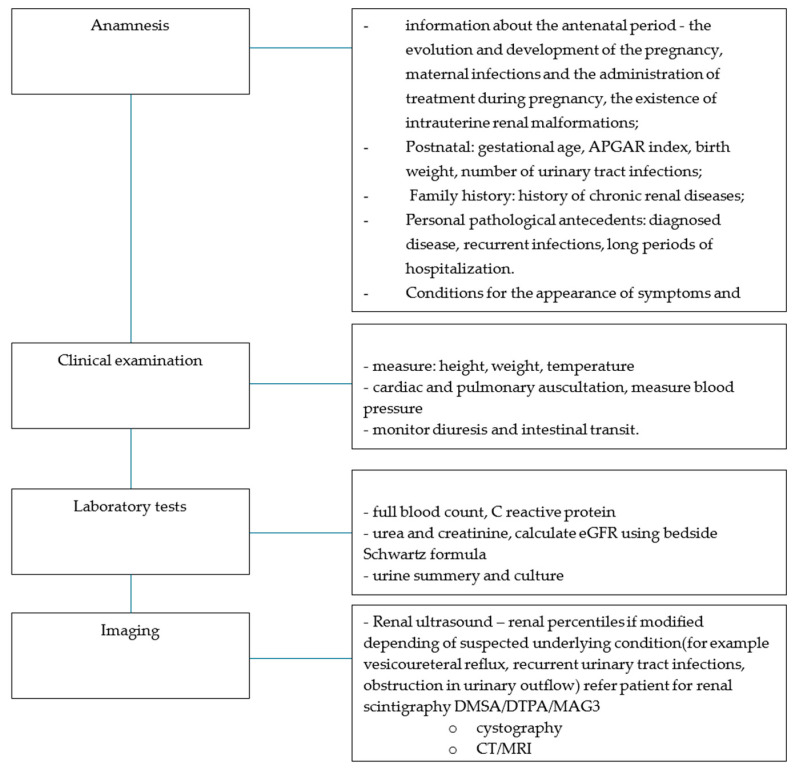
Proposed algorithm for diagnosis and follow-up in patients suspected of CAKUT for practitioners.

For practitioners, we propose an algorithm for the diagnosis of patients with CAKUT (Figure 6).

## 5. Conclusions

The study highlights the interplay between renal function and structural characteristics. Strong-moderate positive correlations were observed between eGFR and both kidney size percentiles and z-scores. Furthermore, there was a highly significant association observed in obese patients. These findings suggest the utility of cystatin C and creatinine for eGFR together with ultrasound measurements in the pediatric population with CAKUT. A national standardized follow-up plan for patients with CAKUT, which incorporates blood samples and abdominal ultrasound should be implemented.

The economic aspect should be taken into consideration because the cost of determining cystatin C is approximately eight times higher than the cost of determining serum creatinine. Our findings suggest that eGFR in association with renal ultrasound remains a useful and affordable method of monitoring patients with CAKUT.

A multidisciplinary approach of the patients with CAKUT is important, which is why it is important to have a good collaboration between obstetrician, radiologist, family physician, pediatrician, pediatric nephrologist, and pediatric surgeon.

## Figures and Tables

**Table 1:** Baseline characteristics of the population under study.

Characteristic	Sample Size (*n* = 64)
Male gender	38 (59.38%)
Metropolitan areas	35 (54.69%)
Age at diagnosis (months)	12; (2, 60)
Age at the moment of examination (months)	116.50; (65, 180)
Weight at the moment of examination (kg)	30; (18.75, 52.75)
Weight z scores	−0.49 ± 1.80
Height (cm)	131.61 ± 29.36
Height z scores	−0.91 ± 2.01

For a parametric distribution, the results are presented as mean ± standard deviation, whereas for a non-parametric distribution, the results are presented as median and IQR.

**Table 2:** CAKUT types and other associated pathologies.

Anomaly or Pathology (Parameter)	Sample Size (*n* = 64)
Renal malformations	33 (51.56%)
Renal pelvis and ureter malformations	38 (59.38%)
Bladder and urethra malformations	13 (20.31%)
Without vesicoureteral reflux	40 (62.5%)
Vesicoureteral reflux	24 (37.5%)
Right Vesicoureteral reflux	6 (25.00%) *
Left Vesicoureteral reflux	7 (29.16%) *
Bilateral Vesicoureteral reflux	11 (45.83%) *
Renal hypoplasia	19 (29.69%)
Compensatory renal hypertrophy **	13 (20.31%)
Reflux nephropathy **	3 (4.68%)
Cystic dysplasia	8 (12.5%)
Ureteropelvic junction stenosis	15 (23.43%)
Dual-collector system	5 (7.81%)
Unique kidney (congenital/surgical)	4 (6.25%)
‘Horseshoe’ kidneys	2 (3.12%)

*—the percentage is expressed from the patients with vesicoureteral reflux. **—Compensatory hypertrophy is secondary to renal hypoplasia/renal agenesia, and reflux nephropathy is secondary to Vesicoureteral reflux.

**Table 3:** Chronic kidney disease staging according to eGFR and albuminuria.

Parameters	Sample Size (*n* = 64)
CKD G1	40 (62.5%)
CKD G2	8 (12.5%)
CKD G3a	1 (1.56%)
CKD G3b	5 (7.81%)
CKD G4	6 (9.37%)
CKD G5	4 (6.25%)
CKD A1	59 (92.18%)
CKD A2	1 (1.56%)
CKD A3	4 (6.25%)

## Data Availability

The information is contained within this article in its entirety. For additional information, please feel free to inquire with either the original author or the corresponding author. Public access to the data is restricted as a result of the patient privacy standards that regulate the handling of clinical data.
